# Case Report: Endoscopic evacuation of a large cerebellar hemorrhage in a term newborn—a modified approach using an agitation wire enhanced suction device

**DOI:** 10.3389/fsurg.2025.1579852

**Published:** 2025-06-19

**Authors:** C. Wendel, O. Ganslandt, N. Rafat, M. Bittl

**Affiliations:** ^1^ Department of Neurosurgery, Klinikum Stuttgart, Stuttgart, Germany; ^2^ Department of Neonatology, Center for Children, Adolescent and Women’s Medicine, Olgahospital, Stuttgart, Germany

**Keywords:** cerebellar hematoma, term newborn, endoscopic evacuation, neuroendoscopy, minimal invasive, hematoma evacuation

## Abstract

**Background:**

Cerebellar hemorrhage in term newborns is a rare but potentially life-threatening condition requiring prompt intervention. Traditional micro surgical approaches carry significant risks of surgical trauma and post-operative cerebrospinal fluid leakage.

**Case description:**

We present a case of successful endoscopic evacuation of a large cerebellar hemorrhage using a novel suction device with an integrated agitation wire. Complete hematoma evacuation was achieved through a single burr hole approach.

**Conclusion:**

At 12-month follow-up, developmental assessment demonstrated excellent neurological outcome with no evidence of hydrocephalus, suggesting this minimally invasive technique as a viable alternative to traditional approaches.

## Introduction

Cerebellar hemorrhage in term newborns is a rare but potentially life-threatening condition (1, 2). Traditional management includes conservative treatment, external ventricular drainage, or microsurgical evacuation through posterior fossa craniotomy (3, 4). Each approach carries specific limitations: conservative management with or without external ventricular drain risks progressive brainstem compression, while surgery poses significant risks of surgical trauma and post-operative complications, such as hydrocephalus, are common in neonates (5–7).

We present a minimally invasive endoscopic approach using innovative technology for effective hematoma evacuation. The (Stereotactic) Intracerebral Hemorrhage (ICH) Underwater Blood Aspiration technique (SCUBA) allows precise, minimally invasive removal of ICH. A suction device with an integrated agitation wire breaks down clots, for efficient removal, and reduced risk of residual hematoma (8).

## Case presentation

### Birth history

A dichorionic-diamniotic twin male infant with 2,360 g, was born at 37 + 5 weeks gestation via uncomplicated spontaneous vaginal delivery (Apgar scores 8/9/9). The pregnancy was unremarkable with normal antenatal ultrasounds. The infant was delivered without complications. No birth trauma, instrumentation, or coagulation abnormalities were noted in either twin. Family history was negative for bleeding disorders. The fontanel was at level but not bulging.

## Clinical presentation

At 12 hours of life, the infant developed irregular breathing patterns and showed reduced muscle tone plus facial palsy on the left side. Blood gas analysis: pH 7.2, pCO2 21 mmHg, HCO3 12.4 mmol/L, Base excess −20 mmol/L. Blood coagulation tests: The initial Quick value was 18% (INR 3.2), the Prothrombin time (PTT) was 57 s, and the Fibrinogen level was 182 mg/dl. Thereafter, a 54% increase in Quick was observed, with a concomitant decrease in PTT to 29 s and an increase in Fibrinogen to 200 mg/dl.

**Figure 1:**
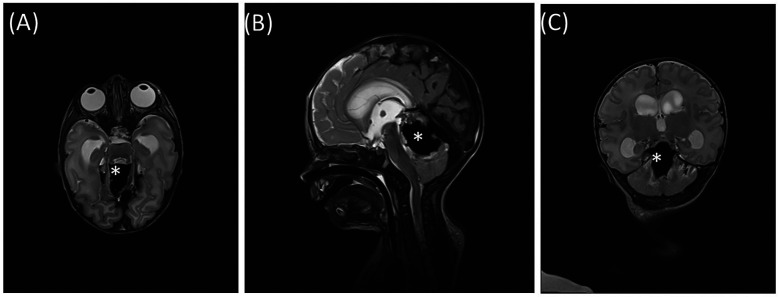
T2—MRI before surgery **(A)** axial, **(B)** sagittal, **(C)** coronal. * Hematoma.

Initial cranial ultrasound demonstrated slightly increased symmetric ventricles. The following magnetic resonance imaging (MRI) (Figures 1A–C) revealed a large vermal cerebellar bleeding (3.0 × 1.8 × 1.5 cm) with compression of the aqueduct. Hydrocephalus was not present (Evans’ index 0.30), but the cerebellum was displaced downwards with tonsillar herniation.

The neonate was then transferred to our neonatal intensive care unit at the first day of life. He was clinically stable but in the following days he showed apneas and developed a hydrocephalus in ultrasounds. A second cranial MRI on day four showed no rebleeding but obstructive hydrocephalus (Evans’ index 0.38).

### Surgical technique

Surgery was performed at day five of life due to progressive neurological deterioration and increase of apneas. Prior to undergoing the surgical procedure, a single fresh frozen plasma concentrate was administered to the patient.

The patient was positioned prone with the head slightly anteflexed on a padded horseshoe headrest. A single burr hole (diameter 10 mm) was placed in the right suboccipital region, 2 cm lateral to the midline and 1 cm below the transverse sinus.

**Figure 2:**
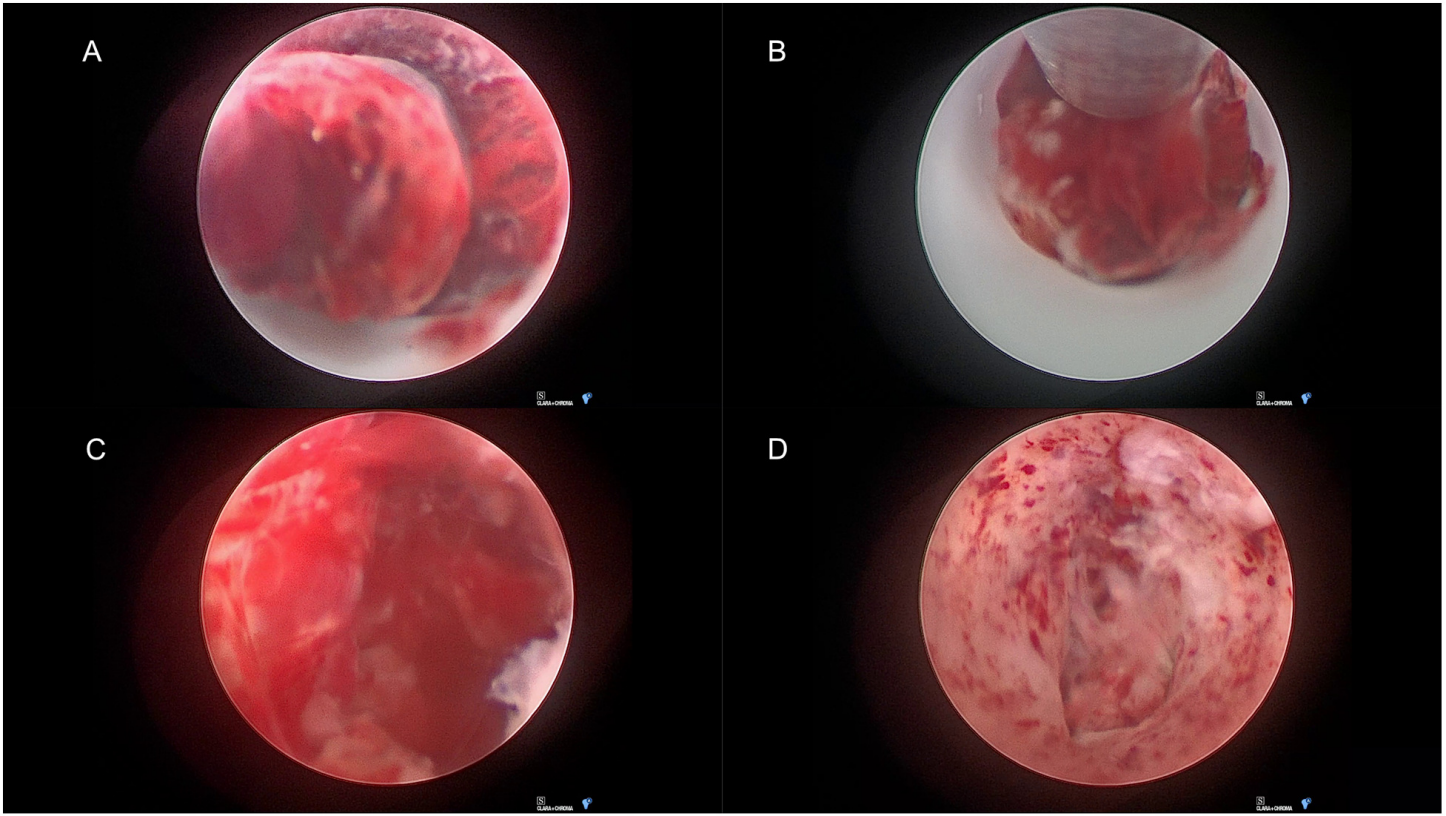
Endoscopic view of cerebellar hematoma. **(A)** Protruding organized hematoma into the peel-away sheet. **(B)** Suction of the hematoma. **(C)** Hematoma cavity with residual blood clot. **(D)** Cavity before completion of surgery.

The endoscopic system consisted of a 6°-degree rigid endoscope with an outer diameter (OD) of 6.1 mm (Lotta, Karl Storz, Germany) inserted through a 19 French peel-away sheet (FH604SU, B. Braun, Germany). A FDA approved suction device with an internal agitation wire was used (OD: 2,8 mm) (Apollo system, Penumbra Inc, Alameda, California). Following the removal of the hematoma, the cavity was irrigated with warmed Ringer's lactate. Inspection of the cavity showed no bleeding sites. (Figures 2A–D) Residual clots were removed. Subsequently, the endoscope was withdrawn, the dura was closed with sutures and a small fibrin onlay patch (Tachosil, Takeda Pharmaceutical Company, Tokyo, Japan). The wound was sutured (Monocryl, Ethicon Inc., Somerville, NJ, USA). Operating time was 93 minutes with an estimated blood loss of 15 ml.

### Imaging outcomes

**Figure 3:**
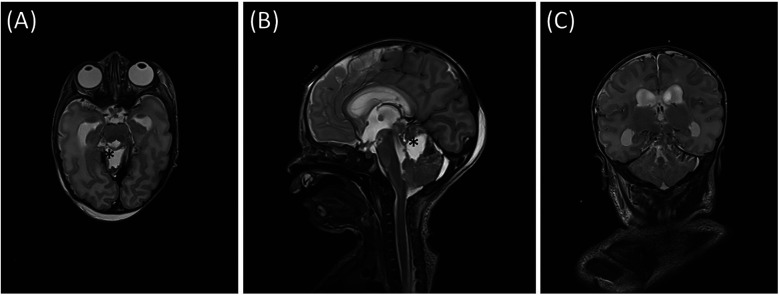
Corresponding planes of T2—MRI after surgery **(A)** axial, **(B)** sagittal, **(C)** coronal. * Hematoma cavity.

Post-operative MRI at 20 hours demonstrated complete evacuation of the cerebellar hematoma, resolution of fourth ventricle compression, as well as decreased ventricular size (Evans’ index 0.35). (Figures 3A–C) Serial cranial ultrasound monitoring showed no development of hydrocephalus in follow up. Pericallosal artery resistance index was 0.77 on postoperative Doppler ultrasound. (Supplementary Material).

### Clinical outcome

The apneic episodes subsided within 24 hours post-surgery. Also feeding improved after surgery. The patient was discharged to the referring hospital on post-operative day 5.

### 12-month follow-up

**Figure 4:**
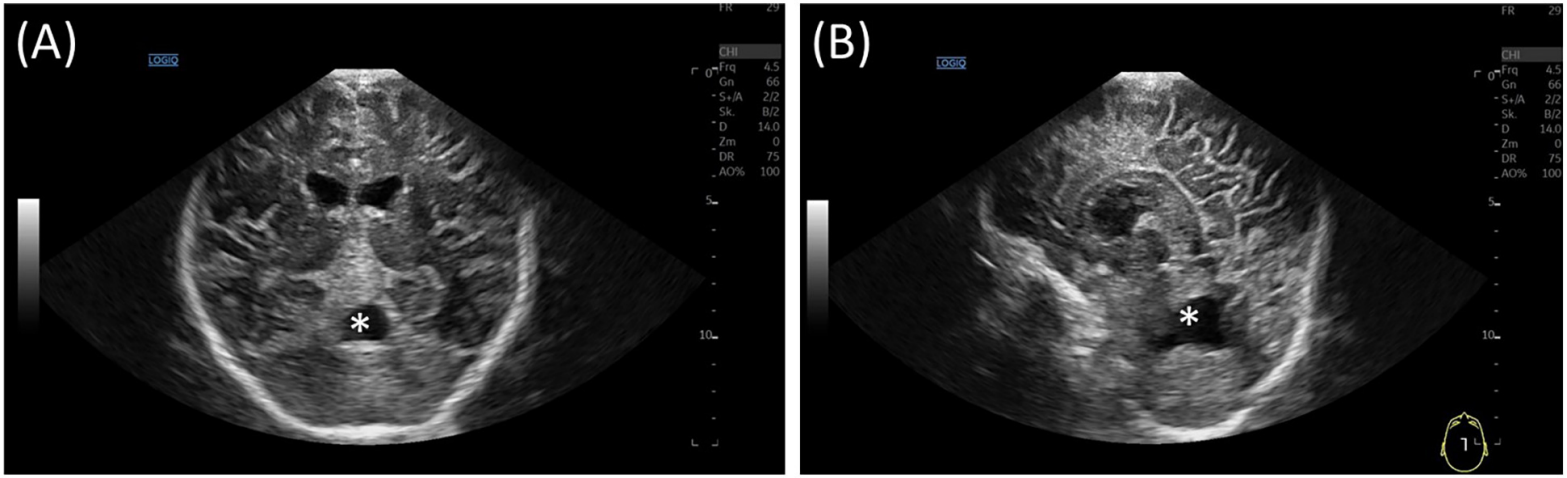
Ultrasound at 12 months follow up, **(A)** coronal view and **(B)** sagittal view. No evidence of hydrocephalus. Cerebellar defect zone in the superior vermis(*).

The infant begins to crawl and to form syllable chains. The infant is able to maintain a passive stance. The hypotonic trunk posture and hypotonic head control when sitting upright indicate a reaction to the right, but not to the left. The patient displays weak triggerable muscle reflexes and strabismus, with no evidence of eye muscle paresis. The head circumference is below the third percentile (43.6 cm). Cranial ultrasound showed a post hemorrhagic defect in the upper part of the vermis (1.4 × 1.7 × 2.4 cm). No signs of hydrocephalus were noted. (Figures 4A,B).

## Discussion

Posterior fossa hematomas in term neonates are often a consequence of prolonged or forceps assisted delivery (3). In most case series, posterior fossa subdural haematomas are the main findings (5, 9, 10).

Large intracerebellar lesions (> 1 cm) in term infants are associated with neurodevelopmental disability (5, 11). Typically, neonatal cerebellar hemorrhage is managed conservatively (4). Alternative approaches to the management of hydrocephalus may include ventricular or ventriculo-subgaleal shunting (3). The primary benefits of this approach are the swift resolution of the hydrocephalus and the relatively minor nature of the procedure. The efficacy and safety of this technique is well-documented; however, the pressure on the brainstem from the blood clot would remain. In cases of brainstem compression and neurological deterioration, urgent surgical evacuation is considered to be the optimal approach, as it is believed to result in a more favorable outcome (3, 12).

In cases where hematoma evacuation is necessary, traditional microsurgical approaches via craniotomy have a proven history. However, surgical route injury and cerebrospinal fluid (CSF) leakage can occur in cases of cerebellar hematomas (13). The endoscopic technique described offers several key advantages:

1. Minimally Invasive: Use of a single burr hole significantly reduces the surgical trauma compared to open surgical methods. This minimizes damage to surrounding tissues and lowers the risk of post-operative complications such as wound infection and CSF leakage (14, 15).
2. Enhanced Visualization: The SCUBA technique provides excellent visualization of the hematoma cavity, allowing for precise evacuation of the clot. Continuous irrigation and controlled suction help maintain a clear operative field (8).
3. Effective Clot Removal: The agitation wire mechanism proves effective in breaking down clot components, facilitating efficient removal and reducing the risk of residual hematoma.

### Outcomes and follow-up

The complete evacuation of the hematoma was confirmed by MRI, with no evidence of surgical route injury or hydrocephalus development. Following cerebellar bleeding, approximately one third of patients need ventricular shunting (3). And In contrast, the present case did not show postoperative hydrocephalus and did not require shunting.

Neurological assessments at 12 months indicated good overall outcomes, although some developmental delays were noted compared to the patient's twin. The patient's head circumference remained below the third percentile, suggesting ongoing monitoring and supportive therapies are necessary.

### Limitations and challenges

1. Successful navigation and use of the endoscopic system require significant amount of training. The cost of the specialized equipment required for this procedure, including the endoscope and suction device, can be a barrier for implementation in resource-limited settings.
2. Optimal candidates are infants with large cerebral hemorrhages (>1 cm), neurological deterioration, hydrocephalus, with no active bleeding or coagulopathy. Careful patient selection is crucial to maximize benefits and minimize risks.

## Conclusion

This article demonstrates a rare case of endoscopic cerebellar hematoma evacuation in a neonate. Endoscopic evacuation using an agitation wire-enhanced suction device is a feasible and effective method for treating large cerebellar hemorrhages in neonates. The positive neurological outcome observed, suggest that this technique may serve as an alternative to traditional approaches, in selected cases where surgery is needed.

## Data Availability

The original contributions presented in the study are included in the article/Supplementary Material, further inquiries can be directed to the corresponding authors.
