# Supplementary figures and images for: Case Report: Endoscopic evacuation of a large cerebellar hemorrhage in a term newborn—a modified approach using an agitation wire enhanced suction device
Source: Front Surg. 2025 Jun 19;12:1579852. doi: 10.3389/fsurg.2025.1579852 (PMC12222118; doi:10.3389/fsurg.2025.1579852)

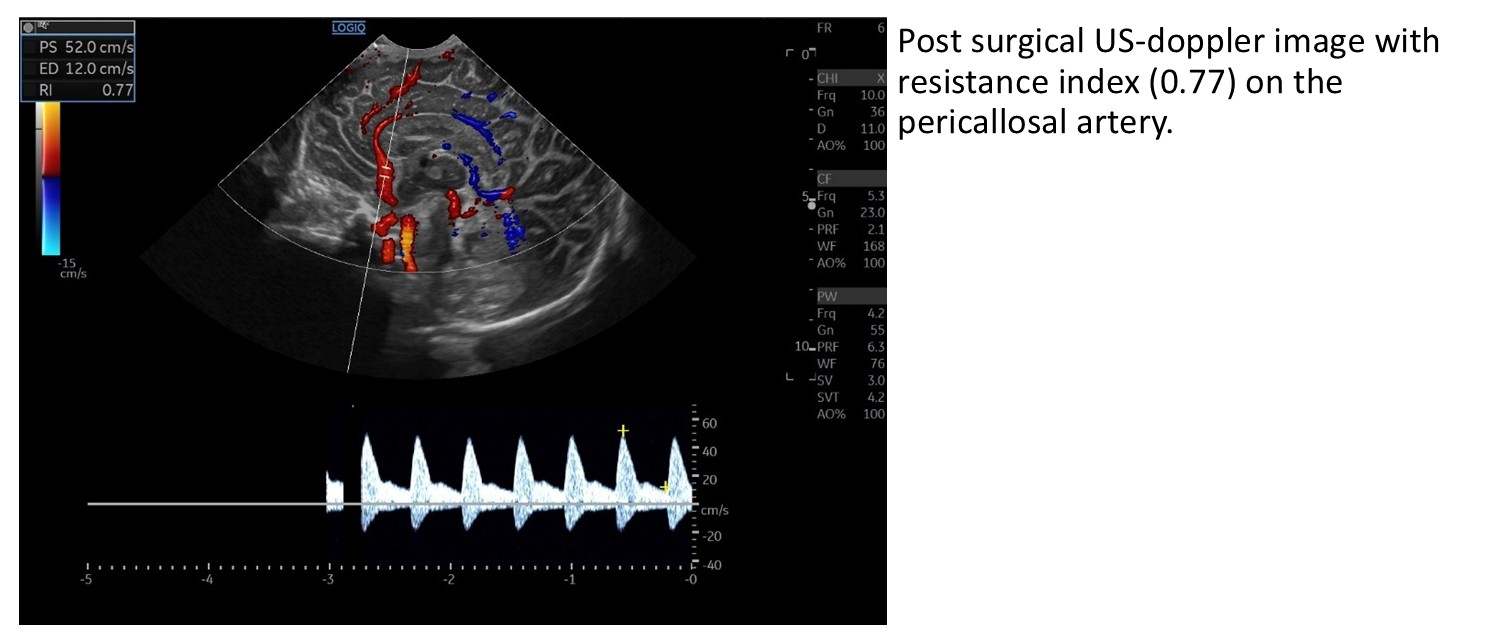

Supplement: Supplementary file 1 [file Image1.jpeg]
